# Supplementary material for: Convergence or dispersion? The impact of China’s innovative city pilot policy on regional innovation differences
Source: PLoS One. 2023 Apr 27;18(4):e0281685. doi: 10.1371/journal.pone.0281685 (PMC10138854; doi:10.1371/journal.pone.0281685)
Supplement: Attachment — Submitted filename: Plos one_response.docx [file pone.0281685.s002.docx]

Gaofeng Wang, Ph.D. Professor

School of Humanities and Social Sciences

University of Science and Technology of China

Hefei, Anhui, 230026, P.R.China

Phone: +86-551-63607920

E-mail: wanggf@ustc.edu.cn

14 December 2022

Dear Editor and Reviewers:

We would like to thank the editor for giving us a chance to resubmit the manuscript, and thank the reviewers for giving us constructive suggestions which would help us to improve the quality of the manuscript. Here we submit a new version of our manuscript with the title “Convergence or dispersion? The impact of China’s innovative city pilot policy on regional innovation differences” (PONE-D-22-22291), which has been modified according to the reviewers’ suggestions. We have separated our responses to the reviewers’ comments according to several categories to achieve an integrated approach in our responses.

Sincerely yours,

Gaofeng Wang, Ph.D. Professor

**Response to editors and reviewers**

1. Literature review and theoretical framework

Response to comment: ***the manuscript lacks a profound theoretical framework.* (***Reviewer #1***); *The literature review section can be improved by adding more relevant studies.(****Reviewer #2****); The*** ***literature review section can be improved by adding more UpToDate studies.(****Reviewer #3****)***

Reply: Thanks to the reviewers for their comments on the literature review and theoretical framework. We put the issue of literature review and theoretical framework under the same category because we think that the lack of theoretical framework is largely due to insufficient review of the existing literature. Therefore, in the literature review part of this manuscript, we add a review of the driving factors of innovation convergence, and further supplement the theoretical explanation of the influence of pilot policy on regional innovation convergence. Specifically, we add to the existing literature exploring the driving factors of innovation convergence, which reveals the role of industry-university-research cooperation (*Hong et al., 2019*), high-tech industrial agglomeration (*Yang et al., 2021*), urban agglomeration strategy (*Tang and Cui, 2021*), high-speed rail (*Yang et al., 2021*), and financial investment in science and technology (*Hou et al., 2022*) in the process of regional innovation convergence. We think that the construction of innovative cities may trigger these mechanisms and thus affect regional innovation convergence. In Section 2.2, we expound on the potential impact of the pilot policy of innovative city on regional innovation differences (convergence) from both positive and negative aspects. In terms of positive impact, the implementation of the pilot policy will accelerate regional innovation convergence through multiple mechanisms, such as increasing government financial investment in science and technology, improving the level of human capital, promoting high-tech industry agglomeration, and deepening industry-university-research collaboration (*Zhou and Li, 2021; Zhang and Wang, 2022*). The rationality of these mechanisms has been confirmed by the newly added references in Section 2.1.2. In terms of negative impact, there are two different interpretations (regional innovation system theory and innovation absorption capacity) of it, which already exist in the content of the original manuscript.

**Related references:**

Hong, J., R. Zhu, B. Hou, and H. Wang. (2019). “Academia-industry collaboration and regional innovation convergence in China.” Knowledge Management Research & Practice 17(4): 396-407.

Yang, N., Q. Liu, Y. Chen. (2021). “Does Industrial Agglomeration Promote Regional Innovation Convergence in China? Evidence from High-Tech Industries.” IEEE Transactions on Engineering Management.

Tang, J., W. Cui. (2021). “Does urban agglomeration affect innovation convergence: evidence from China.” Economics of Innovation and New Technology 1-16.

Yang, X., H. Zhang, S. Lin, J. Zhang, and J. Zeng. (2021). “Does high-speed railway promote regional innovation growth or innovation convergence?.” Technology in Society 64, 101472.

Hou S, He J, Song L. Fiscal science and technology expenditure and the spatial convergence of regional innovation efficiency: evidence from China’s province-level data[J]. Economic Research-Ekonomska Istraživanja, 2022: 1-19.

Zhou, Y., and S. Li. (2021). “Can the innovative-city-pilot policy promote urban innovation? An empirical analysis from China.” Journal of Urban Affairs 1-19.

Zhang, S., and X. Wang. (2022). “Does innovative city construction improve the industry–university–research knowledge flow in urban China?.” Technological Forecasting and Social Change 174, 121200.

2. Explanation of the indicators, the context of the pilot cities, and the method of assessment

Response to comment: ***the authors provide a short list of variables used for assessing the policy impact on pilot cities but they don't elaborate the indicators as such and neither the context of the pilot cities. it's not clear how the authors assess the policy impact on the citites development. there are many more factors driving cities developments than the ones listed. therefore I have strong doubts that the framework presented is suitable for such ambitious analysis.* (***Reviewer #1***)**

Reply: Thank you for your valuable comments, although the description of variables already exists in Section 3.1 of the original manuscript, it is not clear and detailed, just as the reviewer said. In order to clarify the variable selection, we present it in the form of a table (Table 2:Descriptive statistics).

Table 2:Descriptive statistics

| Variable | Variable definitions | Mean | Std. Dev. | Min | Max |
| --- | --- | --- | --- | --- | --- |
| **Explained variables** |  |  |  |  |  |
| innovation index | Measured by Kou | 0.0461 | 1.9376 | -5.2729 | 7.0575 |
| Patent_invention | the number of invention patents granted | 3.8587 | 2.2757 | -6.9077 | 10.3421 |
| Patent _total | the number of all patents granted | 6.4694 | 1.8426 | 0.6931 | 12.3100 |
| **Core explanatory variable** |  |  |  |  |  |
| Policy | dummy variable, with ICP is 1, otherwise is 0 | 0.1158 | 0.3201 | 0 | 1 |
| **Control variables** |  |  |  |  |  |
| Industry_sec | the proportion of secondary industries | 0.4729 | 0.1124 | 0.0276 | 0.9097 |
| n + g+δ | the sum of the natural growth rate (n), technological progress rate (g), and depreciation rate (δ ), where g+δ is equal to 5% | 2.2815 | 0.5164 | -2.9967 | 3.8248 |
| R&D_exp | government fiscal spending on science and technology (10,000 yuan) | 9.3701 | 1.8441 | 3.8712 | 15.5293 |
| H_cap | the number of college students per 10,000 people | 4.4753 | 1.1193 | -0.5242 | 7.1787 |
| Finan | the sum of deposits and loans from financial institutions (10,000 yuan) | 16.7776 | 1.2377 | 13.3523 | 21.1829 |
| C_profit | the total profit of industrial enterprises above a specific size (annual main business income is more than 20 million yuan; (10,000 yuan)) | 5.5071 | 0.9989 | 1.9839 | 8.4233 |
| Commu | the total number of mobile phones and Internet users (10,000 households) | 13.3299 | 1.5551 | 6.5696 | 17.7593 |
| Open | the actual use of foreign capital (USD 10 000) | 9.3026 | 2.6079 | -6.9077 | 14.1523 |
| Trans | the total passenger of transportation (including roads, waterways, and flights; 10,000 people) | 8.4480 | 0.9965 | 0.4055 | 12.5668 |
| **Instrumental variable** |  |  |  |  |  |
| Slope | the average urban slope | 7.9787 | 1.2657 | 4.3791 | 10.0807 |
| Elevation | the average urban elevation (m) | 13.0512 | 1.4005 | 9.5953 | 15.6591 |
| **Other variable** |  |  |  |  |  |
| R&D_talent | The number of scientific research and technical service employees | 8.2991 | 1.0789 | 4.6051 | 13.6048 |

Except for Industry_sec and Policy, all other variables take the logarithm

As for the context of the pilot city, We are sorry for the lack of a clear expression. According to the suggestions of the reviewers, we have added the explanation of specific declaration process (**revised manuscript**: *Currently, to become a pilot city, it must to go through the proper procedure of local recommendation (provincial government), material review, and expert review. Finally, the Ministry of Science and Technology publishes the list of pilot cities, and the project acceptance begins after several years (2-3 years). During this period, pilot cities can explore and formulate development plans according to local conditions.*), and the origin of innovative cities (**revised manuscript**: (*Originated from the practice of Western developed countries in the mid-to-late 1990s, an “innovative city” is the concrete practice of transforming from the traditional investment-driven, development mode to the innovation-driven, development mode. The concept of the innovative city in China can be traced back to the strategic decision of “building an innovative country” made by China State Council in 2006. Subsequently, many cities put pursued the building an innovative city.*). The list of cities in the innovative city pilot program is shown in Table (Table 1).

Table 1:Innovative city pilot list

| Year | Eastern China | Central China | Western China |
| --- | --- | --- | --- |
| 2008 | Shenzhen |  |  |
| 2010 | Beijing (Haidian District), Tianjin (Binhai New District), Shanghai (YangpuDistrict), Dalian, Qingdao, Xiamen, Shenyang, Guangzhou, Nanjing, Hangzhou, Jinan, Suzhou, Wuxi, Yantai, Tangshan, Ningbo, Jiaxing, Shijiazhuang, Changzhou, Fuzhou, Haikou | Changsha, Harbin, Hefei, Taiyuan, Jingdezhen, Wuhan, Nanchang, Luoyang | Chongqing(Shapingba District), Xi’an, Guiyang, Kunming, Chengdu, Baotou, Shihezi, Lanzhou, Nanning, Baoji, Changji, Yinchuan |
| 2011 | Lianyungang, Qinhuangdao, Zhenjiang | Changchun | Xining, Hohhot |
| 2012 | Nantong | Zhengzhou | Urumqi |
| 2013 | Yangzhou, Taizhou, Yancheng, Jining, Huzhou | Yichang, Xiangyang, Pingxiang, Nanyang | Zunyi |
| 2018 | Xuzhou, Quanzhou, Weifang, Longyan, Jinhua, Foshan, Shaoxing, Dongg, Dongying | Zhuzhou, Hengyang, Jilin, Ma’anshan, Wuhu | Yuxi, Lhasa, Hanzhong |

**As for the rationality of method and variable selection, the methodology of our study builds on existing innovation policy evaluations.** Many studies selected specific policy shocks to construct quasi-natural experiments and investigated the causal relationship between innovation policy and innovation performance using the policy evaluation method of difference-in-differences (DID). For example, based on data from 241 cities in China from 2005 to 2017, *Li et al.(2022)* took innovative city construction as a "quasi-natural experiment" and used the difference-in-differences method to empirically study the impact of China's innovative city construction on urban green innovation. *Zhang and Wang (2022)* used the spatial difference-in-difference model to investigate the effect of innovative city pilot policy on knowledge innovation efficiency and knowledge transformation efficiency. Relying on the synthetic control method and difference-in-differences, *Robbiano (2022)* took the establishment of the Italian Institute of Technology (IIT) as a policy change useful to understand the causal effect of public-funded research centre on the regional innovation capacity. Similarly, based on the data of 281 cities from 2005 to 2016, *Zhou and Li (2021)* constructed difference-in-difference (DID) models to study the impact of national innovative-city-pilot policy on urban innovation.

The principle of DID is to use observation data to simulate experimental research design. Its basic idea is to divide the survey samples into two groups: the group affected by policy, namely the experimental group, and the group not affected by policy, namely the control group. Firstly, the change amount of an index in the experimental group before and after the policy is calculated, and then the change amount of the same index in the control group before and after the policy is calculated, and then the difference of the above two variables is calculated, so as to reflect the net impact of the policy. **According to this principle, this manuscript divides cities into pilot cities and non-pilot cities based on the pilot policy of innovative city in China, so as to evaluate the effect of innovative cities (influence on regional innovation convergence).** As control variables for this paper, including the industrial structure, natural growth rate, technological progress rate, government fiscal spending on science and technology, traffic conditions, development of enterprises and financial institutions, the level of human capital, communication and opening to the outside world, are also selected based on existing studies. (*Zheng and Li, 2020; Yang et al., 2021; Tang and Cui, 2021; Yang et al., 2021*).

**Related references:**

Kou, Z. L; and X. Y. Liu. (2017). “FIND Report on City and Industrial Innovation in China (2017).” Fudan Institute of Industrial Development, School of Economics, Fudan University.

Zheng, S., and Z. Li. (2020). “Pilot governance and the rise of China's innovation.” China Economic Review, 63, 101521. <https://doi.org/10.1016/j.chieco.2020.101521>.

Zhang, S., and X. Wang. (2022). “Does innovative city construction improve the industry–university–research knowledge flow in urban China?.” Technological Forecasting and Social Change 174, 121200. https://doi.org/10.1016/j.techfore.2021.121200.

Yang, N., Q. Liu, Y. Chen. (2021). “Does Industrial Agglomeration Promote Regional Innovation Convergence in China? Evidence from High-Tech Industries.” IEEE Transactions on Engineering Management <https://doi.org/10.1109/TEM.2021.3084936>.

Tang, J., W. Cui. (2021). “Does urban agglomeration affect innovation convergence: evidence from China.” Economics of Innovation and New Technology 1-16. <https://doi.org/10.1080/10438599.2021.1973896>.

Yang, X., H. Zhang, S. Lin, J. Zhang, and J. Zeng. (2021). “Does high-speed railway promote regional innovation growth or innovation convergence?.” Technology in Society 64, 101472. <https://doi.org/10.1016/j.techsoc.2020.101472>.

Yang J, Xiong G, Shi D. Innovation and sustainable: Can innovative city improve energy efficiency?[J]. Sustainable Cities and Society, 2022, 80: 103761.

Robbiano S. The innovative impact of public research institutes: evidence from Italy[J]. Research Policy, 2022, 51(10): 104567.

Li L, Li M, Ma S, et al. Does the construction of innovative cities promote urban green innovation?[J]. Journal of Environmental Management, 2022, 318: 115605.

3. The definition of convergence and dispersion

Response to comment: ***What is meant by convergence or dispersion in this study?* (***Reviewer #2***)**

Reply: Thanks to the reminding of the reviewer. We have put the definitions of convergence and dispersion in Section 2.1.1, the details are as follows:

*Convergence hypothesis refers to when the economic growth rate of latecomer regions grows more quickly than the advanced one, and the per capita income level of latecomer regions converges with that of advanced regions in the long run. Similarly, convergence in the context of innovation refers to innovation production in latecomer regions that have a faster growth rate, and the innovation gap between regions tends to converge. On the contrary, dispersion means that the growth rate of innovation is slower in the latecomer regions, and the gap tends to widen.*

**Related references:**

Castellacci, F., and D. Archibugi. (2008). “The technology clubs: The distribution of knowledge across nations.” Research Policy 37(10): 1659-1673.

Barrios, C., E. Flores, and M. Á. Martínez. (2019). “Club convergence in innovation activity across European regions.” Papers in Regional Science, 98(4): 1545-1565.

4. The reason that China is chosen for the context

Response to comment: ***Why China is chosen for the context of this study?* (***Reviewer #2***)**

Reply: Thank you for your valuable comment. Please allow me here to make a brief explanation of the selection of China as the research background. What we first observed was regional innovation differences in China. In a few decades, while China achieved remarkable innovation development, innovation activities have shown significant regional imbalances. **Therefore, the first reason is that China has the need for coordinated development of regional innovation.**

In contrast, the growth rate reflects the possibility of bridging gap between cities. Figure 1 of the manuscript also shows this phenomenon. In the process, as one of the determinants in regional innovation system, the role of government at all levels in shaping regional patterns cannot be ignored. Owing to the particularity of China's system, the coordinated interaction between central and local innovation practices has become an important experience and characteristic of China's implementation of innovation-driven development strategies and the construction of an innovative economy. Therefore, we cannot help but ask whether China's late-developing regions are really converging with pioneering regions under the influence of national innovation policy. **Here, the second reason we classify as the characteristics of Chinese government functions**. As a typical example of a country with strong government intervention, It's worth exploring whether the possibility of bridging gap between cities is related to the government's regional innovation strategy.

Among them, the innovative city pilot is an important exploratory policy for the government to participate in and support urban innovation development. This pilot policy conducted in China provides us with an opportunity to examine the impact of government innovation policy on regional innovation convergence. **So the third reason is that China satisfies the pilot governance premise this study requires.**

4. study's motivation, research question, and contribution

Response to comment: ***The study's introduction requires some improvement to add to the study's motivation, research question, and contribution.* (***Reviewer #2***); *The introduction is well written; however, the research question must be added to clarify the study's objective and what the authors want to achieve from the study's outcome.* (***Reviewer #3***)**

Reply: We are grateful for your comments. According to the reviewer's suggestion, we add to the study's motivation, study's objective, research question, and contribution in introduction. The study's motivation is described as follows:

*As one of the determinants in the regional innovation system, the role of the government at all levels in shaping regional pattern cannot be ignored. Some believe that moderate government intervention can effectively make up for market failures and alleviate the negative impact of innovation resource polarization. Others argue that government intervention may destroy the market mechanism and induce rent-seeking behavior, leading to the loss of innovation efficiency; further exacerbating regional disparities.* ***The differing perceptions of the government’s role suggest that it is vital to clarify the government’s influence when exploring innovation development from a regional perspective****.*

*In this study, we supplement the evidence that government intervention affects regional innovation patterns by examining whether the place-based innovation policy promotes regional innovation convergence, based on the construction of innovative cities in China.*

*…*

*Among them, the innovative city pilot (ICP) is an important exploratory program for the government and supports urban innovation development; prior studies have confirmed its positive effect on innovation and knowledge flow for pilot cities [10-11].* ***However, few empirical studies have examined whether such pilot city programs can narrow regional innovation differences****.…*

We have clarified the main objective and questions of the study：

*The aim of this study is to clarify the role of innovative city construction in regional differences, focusing on the core question of whether the innovative city policy promotes regional innovation convergence. Specifically, considering China’s innovative city pilot program as a quasi-natural experiment, we evaluate the policy from two aspects: whether it stimulates innovation (basic effect) and whether it accelerates innovation convergence (convergence effect).*

The research contribution is also summarized in the penultimate paragraph of the introduction:

*Compared with the extant literature, the contributions of this study are as follows:* ***First****, the research results reveal the basic effect and convergence effect of innovative city construction on regional innovation, which shows the multiple effects of the ICP.* ***Second****, we find that although the construction of innovative cities promotes innovation convergence among pilot cities, it also slows down the overall regional innovation convergence, revealing the dual character of IPC.* ***Third****, following the process of difference-in-differences (DID) method, we conduct a test of the moderating effect based on the β convergence model to better reflect the causal relationship between variables.* ***Fourth****, the temporal and spatial characteristics of the basic effect and the convergence effect are compared, and the regional heterogeneity of the two effects is captured, presenting the interpretation of dual characters regarding ICP.*

As for what we want to achieve from the study's outcome, through the test results of the influence of pilot policies on regional innovation convergence in innovation-oriented cities, we aim to determine whether the regional innovation policies of the Chinese government will exacerbate the existing regional differences, so as to provide empirical evidence for the influence of regional strategy on regional innovation pattern and the direction for the next policy adjustment (For example, whether consideration needs to be given to increase the number of pilot cities in some areas to reduce regional disparities that may widen). The relevant discussion is as follows:

*The research results not only enrich the evaluation research of innovative city construction, but also provides evidence of government intervention to promote the coordinated development of regional innovation from the perspective of pilot governance,* ***which provides a theoretical basis and practical guidance for the government to further summarize the pilot experience and expand the scope of the pilot.***

5. discussion about the study's outcome

Response to comment: ***The results section should be improved by adding more discussion about the study's outcome.* (***Reviewer #2***); *The results section only explains the outcome of the regression models. It is important to discuss these results and why results are positive or negative with economic justification.* (***Reviewer #3***)**

Reply: Thank you for your valuable comments. Based on the suggestions of the reviewers, we have added more discussion in the results section.

① The discussion of Benchmarking results： *After controlling for the factors that potentially affect innovation, the absolute value of the coefficient increases (the speed of convergence accelerates), which means that the growth rate of innovation is not only negatively correlated with the initial innovation level, but also affected by industrial structure, R&D input, and other factors. In columns 3 and 4, after* $\text{Policy}$ *are added, the coefficient of* $\text{L.ln\_Y}$ *increases slightly, indicating that ICP may intensify regional innovation divergence.*

② The discussion of moderating effect results：*Combined with the results of columns 3 and 4 in Table 1, it can be concluded that ICP promotes innovation convergence among pilot cities but intensifies the innovation divergence of the whole sample. This may be the result of a widening innovation gap between the cities undergoing ICP and those that have yet to do so.*

③ The discussion of dynamic effect test results：

*As shown in Figure 4, the basic effect of the ICP has a positive impact for two years following the policy’s implementation. In general, the above comparison demonstrates that the convergence effect of the ICP on innovation development lags slightly behind the basic effect. Considering the small number of pilot cities in the early stage ( in 2008, only one city - Shenzhen, was an ICP ), and it takes a certain amount of time from the start of the pilot (input) to see an increase of innovation output. It is not surprising that both the basic effect and the convergence effect have lag periods.*

④ The discussion of spatial spillover effect test results：

*In general, the results in Table 5 and Table 6 show that ICP has significant innovation spillover effect, which not only improves the innovation level of surrounding cities, but also promotes innovation convergence between these cities and pilot cities. The phenomenon of innovation agglomeration can promote knowledge spillover and technology transfer, so as to achieve further convergence of innovation between cities. However, it is also important to point that the spillover effect is only effective within a certain spatial range..*

⑤ The discussion of regional heterogeneity results：

*Although the western region has a faster convergence rate, ICP does not necessarily significantly promote the convergence between pilot cities in the western region. For many reasons (fewer pilot cities and poorer foundation for innovation), the influence of ICP in western China is not as significant as that in central and eastern China. Even in the western region, there is heterogeneity in the effect of policy implementation among different cities. For Xi’an and Baoji, two cities in the same province in western China, the annual growth rate in the innovation index of the former was 16.4%, much higher than the 6.3% of the latter after the construction of innovation-oriented cities was implemented.*

5. simplification of conclusion

Response to comment: ***The conclusion section is long; it should be short by explaining the key findings and policy implications of the study.* (***Reviewer #2***); *The conclusion part of this research is unnecessarily long, and there are many references. So, it should be short and remove the extra details.* (***Reviewer #3***)**

Reply: Thank you for your valuable comments. Based on the suggestions of the reviewers, we have simplified the conclusion. Specific contents are as follows:

*As one of the determinants in a regional innovation system, the role of government in shaping regional patterns cannot be ignored. The ICP is an important exploratory policy for the government to participate in and support urban innovation development; prior studies have confirmed its positive effect on pilot cities. The conclusions of the empirical research are as follows :*

*(1) Multiple effects of ICP.*

*The innovative city pilot policy not only improves the innovation level of cities (basic effect), but also promotes the innovation convergence among pilot cities (convergence effect).*

*(2) Dual character of ICP .*

*Although the construction of innovative city accelerates the convergence of innovation among pilot cities, it intensifies the dispersion of innovation in the whole region (including pilot and non-pilot cities) in the short-term.*

*(3) Time lag and spatial spillover characteristics of ICP*

*Both the basic effect and the convergence effect have the characteristics of time lag and spatial spillover, but the convergence effect shows a longer time lag and a smaller range of spatial spillover than the basic effect.*

*(4) Regional heterogeneity of ICP*

*Although the western region has a faster convergence rate, the construction of innovative cities does not significantly promote the improvement of urban innovation level and the convergence of innovation between cities.*

Corresponding policy suggestions:

*Based on the above analysis and conclusions, our research has several policy implications.*

*First, given that the pilot policy has both basic and convergent effects, on the premise of giving full consideration to urban innovation resource endowment, development environment and regional pattern, subsequent policy formulation should continue to expand the scope of pilot cities ,while also planning the distribution of pilot cities in different regions and at different levels. The conclusions also apply to other place-based innovation policy pilots; integrating the concept of coordinated development of regional innovation into policy planning to avoid further expansion of innovation differences between regions.*

*Second, due to the limitation of spatial spillover effect in geographical distance, the existing innovative city policy has further widened the innovation gap between the the bottom cities and the top ones. Policymakers should focus on cities that are not yet within a convergent radius. In particular, the sample for this study did exclude some cities, most of which are located in remote western regions. Therefore, we may be underestimating the widening regional innovation differences. For the Chinese government, this is an important challenge for the coordinated development of regional innovation.*

*Third, government should improve the regional innovation synergy mechanism. To promote the convergence of regional innovation by strengthening the radiation effect of innovation center and improving the absorption capacity of marginal area. The new modes to facilitate the cross-city flow of research funds and scientific and technological personnel should be explored. The regional collaborative innovation alliances could also be established to promote closer cross-regional integration of innovation chain and industrial chain and form a regional innovation layout featuring clear main functions, complementary strengths, and high-quality development.*

6. update of data

Response to comment: ***My main concern is about the data. The authors used data from 2003-2016, which is old. The current year of 2022 is near to end, so how can authors give policy implications based on these results? So, I suggest adding up-to-date data and revising the analysis.* (***Reviewer #2***)**

Reply: Thank you for the caution. In the original manuscript, we used data from 2003 to 2016, largely because the innovation index measured by Kou was only updated to 2016. In the revised version, we supplement the innovation Index for 2017 to 2020 in terms of relative ratios (the ratio of the current year to the previous year) based on the city's invention patent grant data. It should be pointed that all of the above analyses are based on updated data.

7. References and pictures

Response to comment: ***This manuscript has many*** ***figures, so authors should keep the important ones and delete the rest.* (***Reviewer #3***). *The introduction has more than 20 references; I think it should be reduced.* (***Reviewer #3***)**

Reply: Thanks for the reminding of the reviewer. We have deleted some figures and references to simplify the manuscript.
